# Green TFP Heterogeneity in the Ports of China’s Pilot Free Trade Zone under Environmental Constraints

**DOI:** 10.3390/ijerph182412910

**Published:** 2021-12-07

**Authors:** Zongbiao Hu, Feng Lan, Han Xu

**Affiliations:** 1 Department of Economics and Trade, School of Business Administration, Zhongnan University of Economics and Law, 182 Nanhu Avenue, Wuhan 430073, China; zbhu@zuel.edu.cn (Z.H.); lannnnnfeng@foxmail.com (F.L.); 2 Department of Business Administration, School of Management, Wuhan College, 333 Huangjiahu Avenue, Wuhan 430212, China

**Keywords:** pilot free trade zone, port, green TFP, environmental constraints, regional heterogeneity

## Abstract

In the context of China’s Pilot Free Trade Zone (FTZ), ports have a new opportunity to realize high-quality development. Based on the analysis of the current situation of pollutant emissions from ports in China’s Pilot Free Trade Zones (FTZs), this paper introduces environmental factors into the analysis framework of the total factor productivity (TFP) of ports in China’s FTZs, and uses the Global Malmquist–Luenberger index method to analyze the evolution trend and heterogeneity of green TFP in 28 ports of China’s 19 FTZs from 2011 to 2017. The results show that firstly, the emissions of sulfur dioxide (${\mathrm{SO}}_{2}$), nitrogen oxides (${\mathrm{NO}}_{X}$) and other pollutants in China’s FTZs have been decreasing year by year. Secondly, both the green TFP and the traditional TFP of the ports in FTZs are on the rise. The absence of environmental factors leads to the underestimation of the TFP of ports. For the green TFP, the main source of its growth is technological progress. Thirdly, there is obvious port heterogeneity in the green TFP of FTZ ports. Nanjing Port has the highest green TFP growth rate, with an average annual growth rate of 21.95%. Ningbo Port, which ranks 14th, has an average annual growth rate of 5.46%. Fuzhou Port, which is rated last, has negative growth. Fourthly, there is also obvious types and regional heterogeneity in the green TFP of FTZ ports. When categorized by type, the average annual growth rate of green TFP in inland ports is significantly higher than that of coastal ports. When categorized by region, the descending order of the average annual growth rate of green TFP is the western region, the eastern region and the central region. Fifthly, the green TFP differences among the eastern, central, and western regions, as well as between inland ports and coastal ports, are shrinking. Moreover, the green TFP differences within inland ports and coastal ports and within central ports and eastern ports are also shrinking, implying there may be σ convergence. The conclusions of this paper have important implications for the scientific understanding of the heterogeneity of green TFP growth in ports in China’s FTZs, and how to promote the green development of ports in China’s FTZs under environmental constraints.

## 1. Introduction

With the continuous strengthening of economic globalization, the impact of the eco-nomic crisis is more likely to spread all over the world [1]. Affected by the economic crisis to varying degrees, the economic growth trend of developed countries and emerging economies are gradually divided. To maintain its dominant position in the global eco-nomic system, regional free trade agreements such as bilateral investment agreements, Trans-Pacific Partnership Agreements, Transatlantic Trade and Investment Partnership Agreements led by developed countries continue to emerge. Compared with the multilateral trade rule system, the economic conditions and needs of countries in the same region are more similar, and regional free trade agreements dominated by bilateral agreements are easier to achieve. Therefore, more and more countries and regions began to sign regional free trade agreements and establish free trade areas (FTA). Consequently, bilateral and free trade pilot areas expanded rapidly in quantity and depth [2]. In recent years, the decline of China’s exports has had an impact on the domestic economy; the growth of domestic demand is slow, the contradiction of overcapacity is prominent, and the structural transformation has a long way to go. China’s economy urgently needs a “booster”, to improve China’s economic recovery and move towards a new stage of a higher-energy level. In this context, in order to meet the urgent needs of economic globalization and regional economic integration, and to achieve the goal of high-quality and sustainable economic growth, China’s pilot free trade zone (FTZ) was materialized.

Unlike the international traditional free trade area (FTA), which focuses on multi-national participation and in which rules are jointly formulated by many countries, a FTZ is a regional special economic zone, established in its own country (region) according to its own (regional) laws and regulations. FTZ strategy requires institutional innovation as the core, accelerating the construction of an institutional framework in line with the general rules of international investment and trade, and accumulating experience for China to gain more voice in international economic cooperation. It is an important strategy for China to actively integrate into the world economic development trend, improve international competitive strength and actively participate in global governance. With the establishment of FTZ, the port, as one of the cores of FTZ, has developed rapidly. According to the statistics of the National Bureau of Statistics of China, the cargo throughput of coastal ports above the designated size increased from 6.16 billion tons in 2011 to 9.19 billion tons in 2019, and the number of 10,000-ton berths of inland ports above the designated size also increased from 340 in 2011 to 444 in 2019, showing rapid development in terms of volume. However, the increase in port input and output does not equate to the improvement of port efficiency. The low efficiency of ports in FTZs and the lack of market competitiveness may still exist. In the context of China’s supply-side structural reform, emphasizing high-quality development, it is contrary to the development concept to promote the port development in FTZs by relying only on the input of production factors. Therefore, incremental reform should be used to promote the stock adjustment, correct the distortion of port factor allocation, and promote development by relying on the improvement of total factor productivity (TFP).

At the same time, China’s FTZ ports adhere to the concept of green development in the development process. As one of the five development concepts first proposed at the fifth plenary session of the 18th CPC Central Committee, the green development concept emphasizes taking a green low-carbon cycle as the main principle, and ecological civilization construction as the basic starting point of development. This is undoubtedly significant for the sustainable development and high-quality development of ports in FTZs. In fact, as early as November 2002, the report of the 16th CPC National Congress put forward the specific objectives of ecological construction, which emphasized the protection of the environment and resources as the basic national policy, and began to realize that “good ecology” is one of the symbols of “civilization”. The report of the 17th CPC National Congress put forward the scientific concept of “ecological civilization”, which outlined that “establishing the concept of green and low-carbon development, focusing on energy conservation and emission reduction, improving the incentive and restraint mechanism, accelerating the construction of resource-saving and environment-friendly production and consumption modes, and enhancing the ability of sustainable development” was taken as the general requirement for the development of the national economy. In the report of the 18th CPC National Congress, China incorporated the construction of ecological civilization into the “five-pronged strategy” (economic, political, cultural, social and ecological progress), established the status of a “long-term plan” for the construction of ecological civilization, and clarified its internal relationship with economic construction, political construction, cultural construction, social construction. On this basis, the construction of ecological civilization was highlighted again in the report of the 19th CPC National Congress, which proposed to “establish and improve the economic system of green and low-carbon circular development, build a market-oriented green technology innovation system, develop green finance, expand energy conservation and environmental protection industry, clean production industry, clean energy industry, and build a clean, low-carbon, safe and efficient energy system”. It can be seen that the concept of green development has been consistently emphasized in economic development, including FTZ ports.

However, although China’s FTZ ports adhere to the concept of green development and promote sustainable development, the operation of port ships still brings much air pollution. In the era of low-carbon economy and green cycle sustainable development, reducing energy consumption and pollution emissions is the top priority of port construction, and also an important standard to evaluate the efficiency of port operation. Moreover, in the “ecological construction” of the 14th five-year plan, it is also emphasized that both economically developed and underdeveloped regions must take “green development” as the precondition of a series of construction requirements, and follow the road of sustainable development unswervingly. Therefore, in the process of promoting economic development through FTZ port development, the concept of green and sustainable development should also be upheld, to reduce the impact on the environment as much as possible. Achieving the green development of ports in FTZs is a difficult problem for China in developing FTZs, and one of the most effective ways to solve this problem is to improve the green TFP of ports. In this context, the main research question of this paper is of the changing trends and heterogeneity of green TFP of the ports in China’s FTZs, from the perspective of environmental constraints. Based on this research question, this paper mainly includes three research purposes: (1) analyze the current situation of the port environment in China’s FTZs by using pollution emission data such as ${\mathrm{SO}}_{2}$ and ${\mathrm{NO}}_{X}$; (2) introduce environmental factors into the measurement framework of the TFP of the ports in FTZs, to measure green TFP and compare it with traditional TFP; (3) empirically analyze the port heterogeneity, type heterogeneity and regional heterogeneity of TFP changes, as well as the evolution trend characteristics of port and regional differences. This study is not only conducive to the assessment of the performance of green growth in the ports of FTZs, but also conducive to the analysis of regional differences and evolution trends of green TFP growth in the ports of FTZs. It is of great practical significance for China to formulate appropriate policies for the development of ports in FTZs, to promote the green and sustainable development of ports in FTZs.

The main conclusions of this paper are as follows: first, pollutants such as ${\mathrm{SO}}_{2}$ and ${\mathrm{NO}}_{X}$ emitted by ports in China’s FTZs are decreasing year by year, but there are heterogeneous characteristics in pollutant emissions that change these trends. Second, the green TFP and traditional TFP of ports in FTZs are rising. Introducing environmental factors will have a significant impact on the estimation of the TFP of ports in FTZs; thereby, not introducing environmental factors will lead to the underestimation of TFP on the whole. The main source of traditional TFP growth is technical efficiency, and green TFP is technical progress. Third, there is great port heterogeneity in the growth of green TFP in the ports of FTZs. Nanjing Port has the highest green TFP growth rate, with an average annual growth rate of 21.95%. Ningbo Port which ranks 14th has an average annual growth rate of 5.46%. Fuzhou Port, which is rated at the bottom, has negative growth. After the introduction of environmental factors, the green TFP of most ports (except Fuzhou Port, Harbin Port and Yangpu Port) has increased, compared with the traditional TFP, and the main source of growth has changed from technical efficiency to technical progress. Fourth, the green TFP growth of FTZ ports also has large range of types and regional heterogeneity. Taking the type as the division standard, the average annual growth rate of green TFP for inland ports is significantly higher than for those of coastal ports. Taking the region as the division standard, the average regional annual growth rates of green TFP in descending order are: the western region, the eastern region and the central region. Fifth, the green TFP differences among the eastern, central, and western regions, as well as between inland ports and coastal ports, are shrinking. Moreover, the green TFP differences within inland ports and coastal ports, and within central ports and eastern ports, are also shrinking, implying there may be σ convergence.

## 2. Literature Review

During the 12th five-year plan period, China proposed the strategic adjustment of economic development, and to pay attention to the deep-seated structural problems of the economy. Therefore, during 2011–2015, China’s economic development entered a period of deep adjustment, and the economic growth rate has changed from the previous high-speed growth to medium and low-speed growth. For China, higher requirements were put forward in terms of economic structure transformation and the degree of opening to the outside world [3]. China’s new stage of “opening-up” is essentially a transition from the border opening-up, to domestic institutional opening-up. The consequent expansion of this opening-up through service trade promotes domestic institutional reform [4]. Therefore, the implementation of the FTZ strategy is the “second quarter” of China’s open economy [5]. The main types of FTZs are entrepot distribution type, trade–industry combination type, export-processing type, and bonded-storage type. Different types of FTZs have played an important role in the process of China’s foreign trade liberalization [6]. These different types of FTZs are also the basis for the construction of a new round of FTZs in China [7,8,9].

Overall, the implementation of the FTZ strategy is conducive to accelerating the pace of China’s opening to the outside world, optimizing the foreign trade structure, and strengthening the economic and trade relations with other countries [10,11,12]. The establishment of FTZ is conducive to reducing regional transaction costs, and legally filtering the political forces of trade protection [13,14]. It also plays an important positive role in promoting the construction of the Belt and Road Initiative [15]. However, without an effective coordination mechanism, this positive role will also be affected [16,17]. Previous studies have shown that FTZs in different regions have different policy effects on promoting economic growth, upgrading industrial structure and capital flow [18,19,20,21,22]. In order to further achieve high-quality economic development through the construction of FTZs, we must make full use of the relationship between FTZs and various industries, policies and related infrastructure. Vigorously exploring FTZs is conducive to strengthening the role of FTZs in serving national strategies [23,24,25].

Scholars have also carried out multi-dimensional research on ports, mainly including the following aspects: first, the research on port cities, such as the port city evolution model [26,27], port city interactive development [28,29], port city interface and spatial planning [30]; second, the research on port logistics, such as the relationship between port logistics and regional economy [31], and port logistics efficiency evaluation [32,33]; third, the research on the port system, such as the evolution model of the port system [34,35] and the impact of new technologies on the port system [36]; fourth, the research on port hinterland, such as the division of port hinterland, and the port hinterland relationship with evolution law [37]; fifth, the research on port transportation, such as the complexity of the shipping network [38,39], and spatial connections and regional differences of the shipping network [40,41]. Among them, the research on port logistics efficiency evaluation is most relevant to this paper.

At present, there are many evaluation methods for port efficiency, which are mainly divided into nonparametric and parametric methods. Data Envelopment Analysis (DEA) is one of the most widely used nonparametric methods. Seth and Feng [42] used the DEA method to evaluate the efficiency changes of 15 container ports in the United States. Barros [43] introduced the Malmquist index on the basis of the DEA to analyze the technical efficiency of five Portuguese seaports in 1999–2000. González et al. [32] used the DEA–Malmquist index to calculate the changes to port efficiency in Spain from 2011 to 2018. Wu and Liang [44] and Li et al. [45] also used the same method to calculate the port efficiency in different regions. Wang and Meng [46] compared the efficiency of China’s inland and coastal ports based on the meta-frontier and the sequential SBM-DEA method, and concluded that the overall efficiency of China’s ports was low. Some scholars also calculate the port efficiency from the perspective of port managers (port-listed companies) [47].

In recent years, more and more scholars have begun to pay attention to the environmental problems caused by port development. When examining port development, environmental factors are often introduced to measure whether green and sustainable development has been achieved. Teixeira et al. [48] carried out a bibliometric and a structured analysis of the literature focusing on the connection between lean, green, and sustainability concepts. Ikram et al. [49] constructed a corresponding green assessment framework for the goal of sustainable development. Lin et al. [50] used the MDAM model to evaluate the improvement strategy of green infrastructure to support urban sustainable environmental construction. Parida [51] and Ranjbar et al. [52] discussed the interactive relationship between green building and the sustainable development of the environment. Xie and Zhu [53] studied how manufacturing enterprises broke through the dilemma of system and efficiency with the help of green innovation to achieve the sustainable development of enterprises. Scholars have carried out in-depth research on how to incorporate environmental factors into port efficiency measurements in different countries and regions, such as the research on China’s ports by Luo et al. [54], Qi et al. [55], and Liu et al. [56], and the research on European and Spanish ports by Quintano et al. [57] and Tovar and Wall [58]. In the study of China’s ports, Luo et al. [54] used the SBM-DEA model to set the annual CO_2_ emissions of China’s eight major container ports as undesirable output to evaluate their environmental efficiency. Qi et al. [55] used the RAM-DEA model to empirically test the statistical data of 10 coastal ports in Jiangsu Province and evaluated the port efficiency from two aspects of port operation performance and environment. Liu et al. [56] refined the research perspective to the ports of FTZs, took the air pollution emitted by ship operation as the undesirable output of the ports and combined this with the expected output to measure the port efficiency. However, they only considered the coastal ports of FTZs, and did not do in-depth research on the changing trends and convergence degrees of green TFP. In the research on ports in other countries and regions around the world, Quintano et al. [57] used the SBM model and REBUS-PLS unit segment detection procedures to detect greenhouse gases such as ${\mathrm{CO}}_{2}$, and ${\mathrm{NO}}_{2}\;$into the output indicators of ports and made a comparative analysis on the ecological efficiency of 24 major container ports in Europe. Tovar and Wall [58] defined ${\mathrm{CO}}_{2}$ as “bad output”, and used the DEA model to calculate the eco-environmental efficiency of port operations of 28 port administrations in Spain, in 2016.

The previous literature has provided the research basis for this study, but there are still some deficiencies in the following aspects. Firstly, although the existing literature has calculated port efficiency, the research on the calculation of the port efficiency of China’s FTZs is still insufficient. With the rapid development of China’s FTZs, it is necessary to study the efficiency of ports in FTZs. Secondly, the existing studies on port efficiency mainly focus on the changes of traditional TFP, and lack research on port green TFP calculation under environmental constraints. Furthermore, port operation has caused serious environmental problems. Therefore, the traditional TFP can’t truly reflect the actual situation of the current port TFP. Thirdly, the existing literature about productivity convergence rarely involves ports. In order to more objectively and scientifically evaluate the differences of TFP among different regions and types of ports in China, it is necessary to further study the convergence or evolution trend of green TFP in ports.

In view of the above problems, the marginal contribution of this study is as follows. Firstly, to conduct in-depth research on the efficiency of ports in China’s FTZs, this paper takes 19 of the current 21 FTZs (except the Shaanxi and Beijing FTZs, due to data availability) as the source of ports, and selects a total of 28 ports as the objects of efficiency measurement. Secondly, we use the Global Malmquist–Luenberger index method to estimate the green TFP of 28 ports in China’s 19 FTZs from 2011 to 2017, and compare this with the traditional TFP without considering environmental constraints. We also study the impact of environmental factors on port efficiency evaluation by comparison. Thirdly, we investigate port heterogeneity, port-type heterogeneity and regional heterogeneity of green TFP on the basis of the overall analysis, and explore the internal causes of the heterogeneity. Fourthly, when analyzing the evolution trend of port green TFP in FTZs, the ports are divided into two types (inland and coastal), and three major regions (east, central and west), and the TFP evolution trend of port types and regional differences are deeply analyzed.

## 3. Analysis on the Current Situation of the Port Environment in China’s FTZs

Since the establishment of China’s first FTZ in 2013, China has established a total of 21 FTZs through “1 + 3 + 7 + 1 + 6 + 3” wild goose array, and gradually expanded from coastal to inland after six rounds of construction. The first batch was the Shanghai FTZ; the second batch included the Guangdong, Tianjin, Fujian FTZs; the third batch included the Shaanxi, Sichuan, Chongqing, Hubei, Henan, Zhejiang, Liaoning FTZs; the fourth batch was the Hainan FTZ; the fifth batch comprised the Heilongjiang, Yunnan, Hebei, Guangxi, Jiangsu and Shandong FTZs; and the sixth batch were the Hunan, Anhui and Beijing FTZs. This means that more than half of China’s provinces have set up FTZs. The new pattern of “no gap along the coast and focus on the mainland” has been formed. Some FTZs are located in the deep inland, with few and small-scale ports (Shaanxi FTZ). Some FTZs have no ports, due to their own special administrative planning (Beijing FTZ), so there are many statistical deficiencies in the ports of these FTZs. Therefore, this paper finally selects 28 ports from 19 FTZs (excluding Shaanxi and Beijing FTZ) as research samples. Before measuring the green TFP and traditional TFP of the ports in FTZs, this paper focuses on the analysis of the environmental pollution of the ports.

### 3.1. Comparison of Pollutant Emission between Inland Ports and Coastal Ports

**Figure 1:**
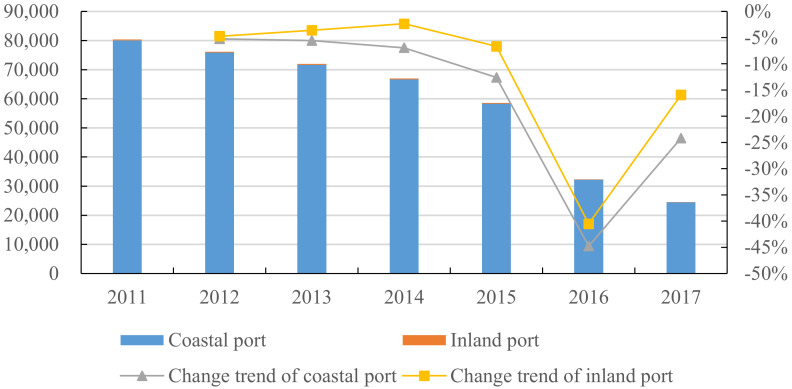
${\mathrm{SO}}_{2}$ emissions from inland and coastal ports (unit: ton). Note: The 2014 ${\mathrm{SO}}_{2}$ emission data of coastal and ocean-going ships and inland ships in the Air Pollutant Emission Inventory of Marine in China (2017) calculate their proportion in China’s ${\mathrm{SO}}_{2}$ emission in 2014 as a fixed proportion value from 2011 to 2017; then, by multiplying by the fixed proportion value according to the ${\mathrm{SO}}_{2}$ emission data in the China Statistical Yearbook and Statistical Yearbooks of each province, the ${\mathrm{SO}}_{2}$ emission data of each port can be obtained.

Figure 1 shows the ${\mathrm{SO}}_{2}$ emissions of inland ports and coastal ports. It can be found that during 2011–2017, the ${\mathrm{SO}}_{2}$ emissions of inland and coastal ports continued to decline year by year, to a certain extent, indicating that the concept of green development has gradually been implemented in the construction and operation of ports in FTZs. According to the comparison of port types, it is not difficult to see that most of the coastal ports in the study sample emit ${\mathrm{SO}}_{2}$, probably because the majority of ocean-going ships in coastal ports emit more ${\mathrm{SO}}_{2}$. From the perspective of the decline rate of ${\mathrm{SO}}_{2}$ emissions, both inland ports and coastal ports have generally shown a process of gradually acceleration and then deceleration. For example, the decline rate sharply increased from 2015 to 2016 and then began to slow down. This shows that the goal of energy conservation and emission reduction has been achieved to a certain extent, and now other measures may be needed to further promote green development.

**Figure 2:**
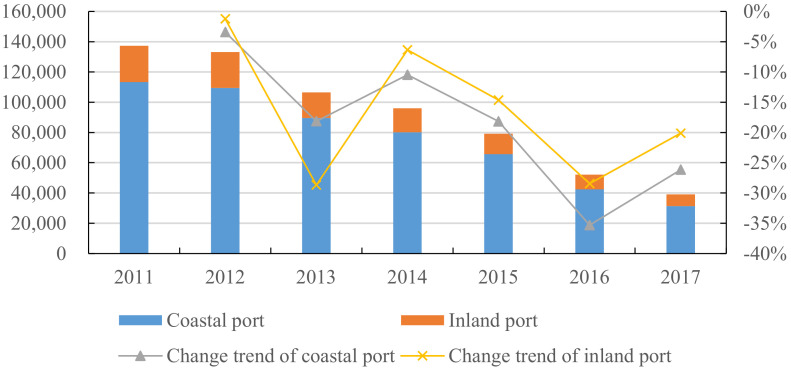
${\mathrm{NO}}_{X}$ emission of inland and coastal port (unit: ton). Note: The 2014 ${\mathrm{NO}}_{X}$ emission data of coastal and ocean-going ships and inland ships in the Air Pollutant Emission Inventory of Marine in China (2017) calculate their proportion in China’s ${\mathrm{NO}}_{X}$ emission in 2014 as a fixed proportion value from 2011 to 2017; then, by multiplying the fixed proportion value according to the ${\mathrm{NO}}_{X}$ emission data in the China Statistical Yearbook and Statistical Yearbooks of each province, the ${\mathrm{NO}}_{X}$ emission data of each port can be obtained.

Figure 2 shows the ${\mathrm{NO}}_{X}\;$emissions of inland and coastal ports. It can be found that the total ${\mathrm{NO}}_{X}$ emissions of ports are decreasing, and that the ${\mathrm{NO}}_{X}$ emissions from coastal ports account for the vast majority of the total emissions; however, compared with the emissions of inland ports, the difference is shrinking. From the range of change, for both the inland ports or coastal ports, the ${\mathrm{NO}}_{X}\;$emission has always been a negative growth, showing a phased downward trend, and the fluctuation degree is basically the same. In addition to 2013, the reduction rate of${\;\mathrm{NO}}_{X}\;$emission in coastal ports was lower than that in inland ports, and it was slightly higher than that in inland ports in other years.

### 3.2. Comparison of Pollutant Emissions among Eastern, Central and Western Ports

**Figure 3:**
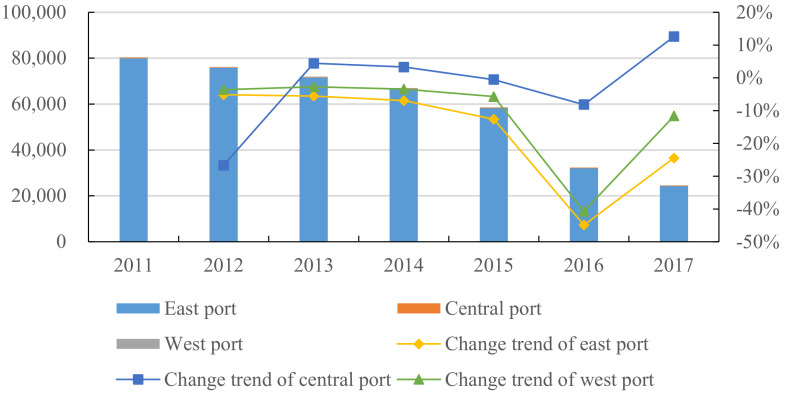
${\mathrm{SO}}_{2}$ emissions from the eastern, central and western ports (unit: ton).

Furthermore, the comparison of pollutant emission in different regions was carried out. Figure 3 shows the${\;\mathrm{SO}}_{2}$ emissions of the ports in East, Central, and West China shows the specific division of ports in the eastern, central and western regions. It can be seen that the eastern ports account for the vast majority of ${\mathrm{SO}}_{2}$ emissions. This is because most of the eastern ports are coastal ports, where ocean-going ships are in the majority and ${\mathrm{SO}}_{2}$ emissions are large. The ${\mathrm{SO}}_{2}$ emissions of eastern ports showed a downward trend, year by year, and the decline rate has remained stable, except for 2016. The variation range of ${\mathrm{SO}}_{2}$ emissions in western ports were basically consistent with those in the eastern ports, but the decrease range was slightly lower than those in the eastern ports. The variation range of ${\mathrm{SO}}_{2}$ emissions in central ports were quite different from those in the other two regions; its ${\mathrm{SO}}_{2}$ emissions increased in 2013, then kept a slow downward trend until 2016, and turned to a positive growth in 2017.

**Figure 4:**
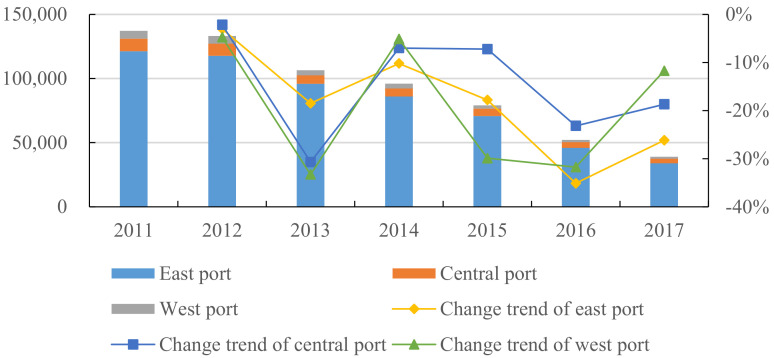
${\mathrm{NO}}_{X}$ emissions from ports in East, Central and West China (unit: ton).

Figure 4 shows the ${\mathrm{NO}}_{X}$ emissions of the ports in the eastern, central, and western regions. It can be seen from Figure 4 that the ${\mathrm{NO}}_{X}$ emissions of the eastern ports account for the vast majority of the total. Under the declining trend of the overall and three regional emissions, year by year, the difference between the ${\mathrm{NO}}_{X}$ emissions of the eastern ports and those of the other two regions has shrunk. From the perspective of the variation trend, ${\mathrm{NO}}_{X}$ emissions of the eastern ports have shown a fluctuating decline, whereas the decline rate of the central and western ports has remained basically the same during 2012–2014. During 2015–2016, the decline rate of the ${\mathrm{NO}}_{X}$ emissions of central ports was significantly lower than those of western ports, which then reversed in 2017. However, the ${\mathrm{NO}}_{X}$ emissions of the two ports have also shown a fluctuating downward trend, as a whole.

To summarize, according to the analysis results of the two major port types of the three regions, the emissions of ${\mathrm{SO}}_{2}$ and ${\mathrm{NO}}_{X}$ from the ports of China’s FTZs have decreased year by year. The port environment is gradually improving, and the concept of green development has been thoroughly implemented. However, the pollutant emission and its variation trend have certain heterogeneous characteristics. Most of the pollutants were emitted from coastal ports and eastern ports, especially ${\mathrm{SO}}_{2}$. On the downward trend, no matter by type or by region, the reduction of ${\mathrm{NO}}_{X}$ emission was higher than that of ${\mathrm{SO}}_{2}$ emission. Therefore, although the pollutant emissions of the ports in FTZs have been decreasing year by year, it is necessary to investigate whether they affect the calculation results of TFP, in the case that there are still a large number of pollutant emissions. Therefore, it is necessary to introduce environmental factors into the study of TFP in FTZ ports for scientific assessment.

## 4. Measurement Methods, Variables and Data

### 4.1. Measurement Methods

#### 4.1.1. Current and Global Production Possibilities Set

First, we needed to build a set of production possibilities, called environmental technologies. This production probabilities set included both “good” outputs, such as GDP, and “bad” outputs, such as ${\mathrm{CO}}_{2}$ emissions. We supposed that in different periods $t\left(t=1,\ldots ,T\right)$, the port $k\left(k=1,\ldots ,K\right)$ used *N* kinds of inputs $x=\left(x_{1},\ldots ,x_{N}\right)\in R_{N}^{+}$ to produce *M* kinds of “good” outputs $y=\left(y_{1},\ldots ,y_{M}\right)\in R_{M}^{+}$ and *I* kinds of “bad” outputs $b=\left(b_{1},\ldots ,b_{I}\right)\in R_{I}^{+}$. For each input vector x, environmental technologies can produce a combination of expected and undesirable outputs simultaneously $\left(y,b\right)$. Based on the hypothesis of Wang et al. [59], we used the data envelopment analysis method (DEA) to convert the current environmental technology into Equation (1):

(1) $$P^{t}\left(x^{t}\right)=\left\{\begin{array}{c} \left(y^{t},b^{t}\right):\sum_{k=1}^{K}z_{k}^{t}y_{km}^{t}\geq y_{km}^{t},m=1,\ldots ,M;\\ \overset{K}{\underset{k=1}{\sum }}z_{k}^{t}b_{ki}^{t}=b_{ki}^{t},i=1,\ldots ,I;\\ \overset{K}{\underset{k=1}{\sum }}z_{k}^{t}x_{kn}^{t}\leq x_{kn}^{t},n=1,\ldots ,N;\\ z_{k}^{t}\geq 0,k=1,\ldots ,K \end{array}\right\}$$

In Equation (1), $z_{k}^{t}$ is the weight measurement index of the observed values of each cross-section, and $z_{k}^{t}\geq 0$ means the constant return to scale. When measuring the GML index, the current production possibility set $P^{t}\left(x^{t}\right)$ was replaced by the global production possibility set $P^{G}\left(x\right)$, which can be expressed as Formula (2) with the DEA method:

(2) $$P^{t}\left(x^{t}\right)=\left\{\begin{array}{c} \left(y^{t},b^{t}\right):\sum_{t=1}^{T}\sum_{k=1}^{K}z_{k}^{t}y_{km}^{t}\geq y_{km}^{t},m=1,\ldots ,M;\\ \sum_{t=1}^{T}\overset{K}{\underset{k=1}{\sum }}z_{k}^{t}b_{ki}^{t}=b_{ki}^{t},i=1,\ldots ,I;\\ \sum_{t=1}^{T}\overset{K}{\underset{k=1}{\sum }}z_{k}^{t}x_{kn}^{t}\leq x_{kn}^{t},n=1,\ldots ,N;\\ z_{k}^{t}\geq 0,k=1,\ldots ,K \end{array}\right\}$$

#### 4.1.2. SBM Directional Distance Function

According to Fukuyama and Weber [60], the global SBM directional distance function incorporated into ${\mathrm{SO}}_{2}$ and ${\mathrm{NO}}_{X}$ emissions on China’s FTZ ports is expressed as:

(3) $$S_{V}^{G}\left(x^{t,k^{\prime }},y^{t,k^{\prime }},b^{t,k^{\prime }},g^{x},g^{y},g^{b}\right)=\underset{s^{x},s^{y},s^{b}}{\max}\frac{\frac{1}{N}\sum_{n=1}^{N}\frac{s_{n}^{x}}{g_{n}^{x}}+\frac{1}{M+I}\left(\sum_{m=1}^{M}\frac{s_{m}^{y}}{g_{m}^{y}}+\sum_{i=1}^{I}\frac{s_{i}^{b}}{g_{i}^{b}}\right)}{2}\;s.\;t.\;\;\sum_{t=1}^{T}\sum_{k=1}^{K}z_{k}^{t}x_{kn}^{t}+s_{n}^{x}=x_{k^{\prime }n}^{t},\forall n;\;\sum_{t=1}^{T}\sum_{k=1}^{K}z_{k}^{t}y_{km}^{t}-s_{m}^{y}=y_{k^{\prime }m}^{t},\forall m;\;\sum_{t=1}^{T}\sum_{k=1}^{K}z_{k}^{t}b_{ki}^{t}+s_{i}^{b}=x_{k^{\prime }i}^{t},\forall i;\;\sum_{k=1}^{K}z_{k}^{t}=1,z_{k}^{t}\geq 0,\forall k;\;s_{n}^{x}\geq 0,\forall n;\;s_{m}^{y}\geq 0,\forall m;\;s_{i}^{b}\geq 0,\forall i$$

In Equation (3), $\left(x^{t,k^{t}},y^{t,k^{t}},b^{t,k^{t}}\right)$ is the input and output vector of ports. $\left(g^{x},g^{y},g^{b}\right)$ is a direction vector, which represents the decrease in input, the increase in “good” output, and the decrease in “bad” output. $\left(s_{n}^{x},s_{m}^{y},s_{i}^{b}\right)$ is a relaxation vector, reflecting the input and output. If the relaxation vectors of both inputs and outputs are positive numbers greater than 0, this means that the actual input and carbon emission of ports are larger than the input-output value of the boundary, whereas the actual output value is smaller than the boundary output value. To summarize, $s_{n}^{x},s_{m}^{y},s_{i}^{b}$ represents the situation of excessive input, relatively insufficient “good” output, and excessive pollution emissions of each port [59].

#### 4.1.3. Global Malmquist–Luenberger Productivity Index

After the construction of the SBM directional distance function, we constructed an output-oriented GML index, to measure green TFP. According to Oh [61], the GML index can be expressed as:

(4) $$GML_{t}^{t+1}=\frac{1+S_{C}^{G}\left(x^{t},y^{t},b^{t};g\right)}{1+S_{C}^{G}\left(x^{t+1},y^{t+1},b^{t+1};g\right)}$$

Furthermore, the GML index can be divided into two parts: the efficiency change index (GEC) and the technology change index (GTC):

(5) $$GML_{t}^{t+1}=\frac{1+S_{C}^{t}\left(x^{t},y^{t},b^{t};g\right)}{1+S_{C}^{t+1}\left(x^{t+1},y^{t+1},b^{t+1};g\right)}\times \left[\frac{\frac{1+S_{C}^{G}\left(x^{t},y^{t},b^{t};g\right)}{1+S_{C}^{t}\left(x^{t},y^{t},b^{t};g\right)}}{\frac{1+S_{C}^{G}\left(x^{t+1},y^{t+1},b^{t+1};g\right)}{1+S_{C}^{t+1}\left(x^{t+1},y^{t+1},b^{t+1};g\right)}}\right]$$

When the $GML_{t}^{t+1}$ (GEC or GTC) index is greater than 1, the green TFP (technical efficiency or technical progress) of the port shows an increasing trend. When the index is equal to (or less than) 1, the green TFP (technical efficiency or technical progress) remains unchanged (or decreases).

#### 4.1.4. Global Malmquist Productivity Index

In order to more intuitively reflect the constraints of environmental factors, such as pollution emissions on China’s FTZ ports, we also estimated the traditional TFP of the ports, and applied the DEA–Malmquist productivity index method (Global Malmquist index method) based on the Global technology, and compared it with the GML index. The Global Malmquist index can be expressed as:

(6) $$GM\left(y^{t+1},x^{t+1},y^{t},x^{t}\right)=\frac{D^{t+1}\left(x^{t+1},y^{t+1}\right)}{D^{t}\left(x^{t},y^{t}\right)}\left[\left(\frac{D^{g}\left(x^{t+1},y^{t+1}\right)}{D^{t+1}\left(x^{t+1},y^{t+1}\right)}\times \frac{D^{t}\left(x^{t},y^{t}\right)}{D^{g}\left(x^{t},y^{t}\right)}\right)\right]=EC\times TC$$

### 4.2. Variable Selection and Data Sources

A total of 21 FTZs have been set up since the first one was established in 2013. Due to the lack of ports in some FTZs or the lack of data in some ports, this paper has collected the input and output data of 28 ports in 19 FTZs in China. The production berth and wharf length are taken as input indicators, the cargo and container throughput as “good output”, and ${\mathrm{SO}}_{2}$ and ${\mathrm{NO}}_{X}$ as “bad output”. The basic data were mainly from the China Port Yearbook, the China Statistical Yearbook and the China City Statistical Yearbook.

#### 4.2.1. Ports Output—“Good Output”

As an important hub of land and water transportation, the port’s basic function is cargo transportation. The existing literature mainly calculates the port efficiency from three perspectives: the input-output perspective of the port itself, the operation perspective of port enterprises, and the consideration of undesirable outputs (Table 1). Among them, most works of literature take the cargo and container throughput as the output indicators, because the cargo and container throughput can directly reflect the output level of the ports. Although some scholars [62,63,64] used financial indicators, such as net profit, main business income and earnings per share, such indicators need to be obtained from the financial data of the ports group, or the ports company that manages the ports. Due to the large number of ports selected in this paper, it is difficult to obtain financial data of some ports. Therefore, the cargo and container throughput were used as the index to measure the “good output” of the ports.

#### 4.2.2. Ports Output—“Bad Output”

At present, there is no unified definition of “bad output” in the academic circle, and the “bad output” used in the measurement of green TFP is also different. In the course of the operation of port ships, ${\mathrm{SO}}_{2}$ and ${\mathrm{NO}}_{X}$ were the two atmospheric pollutants with the largest emissions and the highest pollution degrees. In this paper, they were regarded as undesirable “bad outputs”, and the practice of Liu et al. [56] was used for reference. According to the 2014 ${\mathrm{SO}}_{2}$ and ${\mathrm{NO}}_{X}$ emission data of coastal and ocean-going ships, and inland ships in the Air Pollutant Emission Inventory of Marine in China (2017), the proportion of ${\mathrm{SO}}_{2}$ and ${\mathrm{NO}}_{X}$ in China’s two types of pollutant emissions in 2014 is calculated as the fixed proportion value from 2011 to 2017. Subsequently, according to the emission data of two types of pollutants in the China Statistical Yearbook and the Statistical Yearbooks of each province, multiplied by the fixed proportion value, the ${\mathrm{SO}}_{2}$ and ${\mathrm{NO}}_{X}$ emission data of each port can be obtained.

#### 4.2.3. Ports Input

According to Table 1, from the perspective of the port’s input, the port’s input indicators mainly included the number of berths for production, the length of the wharf, and the amount of loading and unloading equipment [65,66]. These indicators directly reflect the port’s scale and infrastructure construction level; from the perspective of the port enterprise operation, port input indicators focus on financial indicators, mainly including main business cost, number of employees, fixed assets, etc. [62,69]. These indicators can reflect the scale and operation level of port operation companies; from the perspective of undesirable output, energy indicators such as oil consumption and electric consumption can also be selected as port input indicators [70,72]. There are two problems in the actual collection of the latter two types of indicators: first, many ports operating enterprises in FTZs are not listed and their financial status is not disclosed, resulting in great difficulty in data collection of financial input indicators; second, in the process of collecting energy input indicators, it is found that there are many missing values in the relevant data of ports in FTZs. Therefore, based on the principle of data availability, this paper mainly selected the number of berths for production and the length of the wharf as input indicators from the perspective of the port’s own input. The longer the wharf length, the more berths can be built. The more berths for production, the stronger the ship carrying capacity of the port. The two can reflect the infrastructure construction level of the port from the perspective of actual investment, and represent the scale and production capacity of the ports to a large extent. Table 2 reports the descriptive statistical results of each variable.

## 5. Time Series Characteristics and Heterogeneity Analysis of Green TFP in FTZ Ports

### 5.1. Overall Temporal Characteristics

Table 3 reports the TFP index of China’s FTZ ports and their decomposition results. When the environmental factors were not introduced, the annual growth rate of TFP (TFPC) in FTZ ports from 2011 to 2017 was 1.25%, in which the technical efficiency (TEC) increased by 5.95% annually, whereas the technical progress (TPC) showed negative growth, with an annual decline of 4.44%. After the introduction of environmental factors, the annual growth rate of green TFP of the ports in FTZs increased to 4.43%, of which the annual growth rate of technical efficiency decreased to 0.57%. The technological progress showed positive growth, with an annual growth rate of 3.84%. Through comparison, it was found that after the introduction of environmental factors, technical efficiency decreased by 5.38%, whereas the technical progress and TFP growth rate increased by 8.28% and 3.18%, respectively. This indicated that after the introduction of environmental factors, the technological transition and the decreasing factor utilization rate of the port coexist. Although the decline of technical efficiency is large, the improvement of TFP brought by technological progress is enough to counter for the loss caused by the lack of factor utilization. Therefore, the TFP growth rate can still achieve positive growth after the introduction of environmental factors, and the average annual growth rate can be further improved. It can be seen that the TFP estimation of China’s FTZ ports will be affected by environmental factors. Ignoring these environmental factors will lead to the underestimation of the TFP growth rate and technological progress growth rate, and the overestimation of the technical efficiency growth rate.

In addition to 2012, the green TFP and the traditional TFP of China’s FTZ ports have both shown positive growth, which may be due to the fact that since the establishment of the first batch of FTZs in 2013, ports, as an important hub of a region, undertake the functions of trade exchanges and cargo transportation with other countries and regions. To give full play to the role of FTZs in promoting foreign exchanges and economic development, their respective trade zone governments and port groups can pay more attention to port management and technological progress. From 2014 to 2015, the TFP index of the two categories continued to decline, which may be due to the increase in the number of FTZs, or some inefficient FTZ ports pulling down the overall growth rate. On the other hand, it may also be due to the fact that in the early days of the establishment of FTZs, some regions excessively pursued the port’s infrastructure construction and blindly expanded, resulting in repeated construction. In the environment of a shrinking market, there is a lack of overall planning awareness and management capacity, which gives some ports excess capacity and low operational efficiency. Since 2011, the green TFP index has been higher than the traditional TFP index. Combination Färe et al. [73] reveal that the reduction in the “bad” output of ports in China’s FTZs has exceeded the growth rate of the “good” output, and the technological progress rate of ports in China’s FTZs are constantly improving, and moving towards the direction of green development.

According to the decomposition, when it comes to the TFP index, without the introduction of environmental factors, the technical efficiency index is significantly greater than 1, and the technical progress index is less than 1 in most years, showing a negative growth. This indicates that without considering environmental factors, TFP growth mainly depends on the improvement of factor allocation and usage efficiency. By comparison with the results without the introduction of environmental factors, the technical efficiency index decreased significantly after the introduction of environmental factors; on the contrary, the technological progress index, especially in 2016 and 2017, showed significant growth. It may be that under the macro environment of energy conservation and the emission reduction, the RD of new energy and new technology has gradually passed the stage of cost investment, and its dividend has begun to emerge. It can be seen that the introduction of environmental factors has had a crucial impact on the estimation of the TFP of China’s FTZ ports. At the same time, this also reveals that the port efficiency of China’s FTZs has not fully reached its potential of existing resources and technologies, and there is still much room to promote the performance growth of ports by improving their operation and production efficiency.

### 5.2. Investigation of Port Heterogeneity

Table 4 reports the TFP index of each port and its decomposition results. It can be found that the TFP of ports in China’s FTZs are different. Without considering the environmental factors, Hangzhou port has the highest TFP growth rate (with an average annual growth rate of 15.97%), followed by Changsha Port and Luzhou port, and Fuzhou port has the lowest TFP growth rate. After the introduction of environmental factors, Nanjing port has the highest TFP growth rate (with an average annual growth rate of 21.95%), followed by Hangzhou port and Chongqing port. Fuzhou port’s TFP growth rate was still the lowest. From the numerical comparison of the TFP index, we can see that after the introduction of environmental factors, except for Fuzhou port, Harbin Port and Yangpu Port, the TFP index of other ports has increased, with Nanjing port and Xiamen Port increasing by 13.23% and 13.49%, respectively. This also indicates that the TFP ranking of the two ports has significantly improved, ranking first and fifth respectively. Overall, environmental factors have a significant impact on the TFP index of each port.

According to the decomposition term of TFP, for most ports, whether environmental factors are introduced or not determines whether the leading factor of TFP growth is technical efficiency or technical progress. Without the introduction of environmental factors, the technical efficiency index of most ports exceeds the technical progress index (except for Fuzhou port). After the introduction of environmental factors, the comparison of the decomposition terms of most ports is reversed. This shows, once again, that ignoring environmental factors leads to errors in the measurement results of the TFP of ports in China’s FTZs. It also means that there is a large space for the vast majority of ports to further promote the growth of TFP, through the improvement of technical efficiency.

### 5.3. Analysis on Port Types and Regional Heterogeneity

In order to investigate the types and regional heterogeneity of the TFP changes in China’s FTZ ports, the FTZ ports were divided into two types (inland ports and coastal ports) and three regions (eastern, central and western regions). It can be seen from Table 5 that there are great differences in the TFP index and its decomposition terms between inland ports and coastal ports. From the regional point of view, the differences between the eastern ports and the central ports are minor, but there are significant differences between them and the western ports.

Under the classification standard of port types, the TFP annual growth rate of inland ports was significantly higher than those of coastal ports, without the introduction of environmental factors. The TFP annual growth rate of inland ports was 5.61%, whereas for coastal ports this was 1.9%. This difference was mainly determined by technical efficiency. This represents that the difference between coastal ports and inland ports is mainly reflected in factor utilization efficiency, when environmental factors are not considered. It may be that with the opening of the FTZ, coastal areas expand port investment in cases with a more advantageous geographical location, resulting in different degrees of congestion in the number of berths and berth length investment [46]. This leads to low efficiency. After the introduction of environmental factors, the annual growth rate of the TFP of inland ports was still higher than those of coastal ports. Compared with the results without the introduction of environmental factors, the technical efficiency index decreased by different degrees, indicating that in terms of port operation and management, the two types of ports had deficiencies in resource consumption and environmental contamination emission management. Moreover, the technical efficiency of coastal ports had a negative growth. This shows that both inland ports and coastal ports have input in clean technology, green operation and environmental protection technology, and the corresponding input has brought different degrees of income, which lead to the leading factor of TFP growth from the technical efficiency without environmental factors, to the technical progress after the introduction of environmental factors.

Under the regional division standard, without the introduction of environmental factors, the annual growth rate of port TFP from high to low is in the order of western, central and eastern regions. After the introduction of environmental factors, the western region still ranked first, whereas the eastern and central regions alternated. According to the decomposition in terms of the TFP index, compared with the non-introduction of environmental factors, the average annual growth rate of technological progress increased significantly, whereas the average annual growth rate of technical efficiency decreased significantly. Firstly, this shows that the regional ranking of TFP annual growth rate was also affected by environmental factors. Secondly, due to data reasons, ports in the western region solely included Chongqing port and Luzhou port, which may be due to the relatively concentrated population in Chongqing and Luzhou, strong demand for cargo transportation, developed water systems, a long history of ship transportation businesses, rich experience in management and operation, and small pollution emissions from inland ships. Therefore, both traditional TFP and green TFP were ranked first. After the introduction of environmental factors, the ranking of the TFP average annual growth rate in the eastern and central regions alternated, which was mainly affected by the sharp decline in the technical efficiency of the ports in the central region. This reveals that compared with the ports in the eastern region, the ports in the central region still have a certain gap in geographical location, scientific and technological environment, and the concentration of cargo transportation points.

### 5.4. Analysis on Evolution Trend of Port Types and Regional Differences

Since green TFP can better reflect the green growth performance of the ports in FTZs, we only analyzed the change trend of port types and regional differences of green TFP. Referring to the practice of Teng et al. [74], we used the coefficient of variation to measure the degree of regional difference.

**Figure 5:**
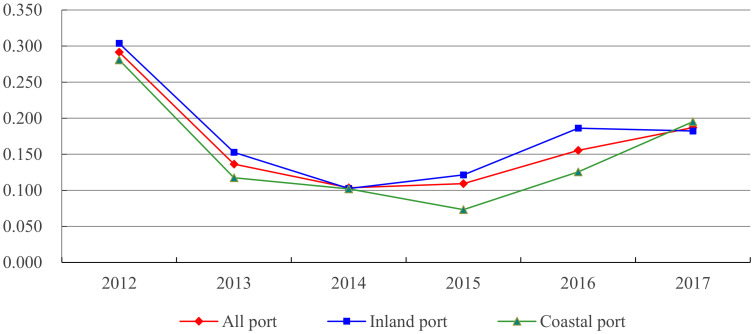
Variation trend of green TFP difference degree in general, inland and coastal ports.

Figure 5 depicts the evolution characteristics of the type difference degree of the green TFP index of the whole sample, inland and coastal ports. It can be found that the difference degree of port type of the green TFP index decreased as time went on, meaning that the green TFP of ports in China’s FTZs may exist on whole σ convergence. From the evolution of the internal differences of green TFP between the two types of ports, the evolution trajectory was basically consistent with the whole sample, which indicated that the differences of green TFP between the two types of ports are shrinking, and may also exist on σ convergence. From the mean value of the coefficient of variation, the inland ports were significantly higher than the coastal ports in most years. Regarding the differences between port types, the difference of coefficient of variation between the two types of ports in 2017 was smaller than that in 2012, indicating that the differences between port types are narrowing.

**Figure 6:**
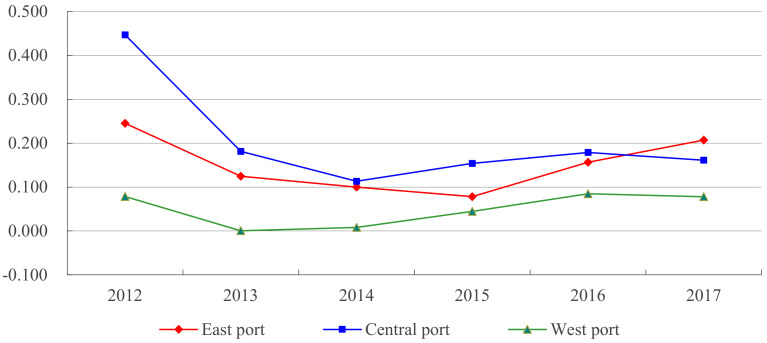
Variation trend of green TFP difference degree in eastern, central and western ports.

Figure 6 depicts the evolution characteristics of regional differences in the green TFP index of eastern, central and western ports. From the evolution of the internal differences of green TFP of ports in the three regions, the coefficient of variation of central ports decreased significantly, from 0.4469 in 2012 to 0.161 in 2017, with the largest declines in 2012 and 2013; the coefficient of variation of eastern ports decreased from 0.2452 in 2012 to 0.207 in 2017, which was less than those of central ports. It can be seen that the differences of green TFP between the central and eastern ports are shrinking, and there may be some problems with σ convergence. However, the coefficient of variation of western ports remained stable during 2012–2017, and there was no convergence trend. From the mean value of coefficient of variation, except for 2017, the coefficient of variation of the three regions from large to small was central, eastern and western, meaning that the difference of green TFP within the central ports was the largest, followed by eastern and western. From the perspective of regional differences, the difference of the coefficient of variation of the three major regional ports in 2017 was smaller than that in 2012, which indicates that the differences between regions are narrowing.

## 6. Conclusions

This paper introduces environmental factors into the measurement framework of TFP in China’s FTZs, and compares it with the traditional TFP, without considering environmental factors. The main conclusions are as follows.

Firstly, the emissions of ${\mathrm{SO}}_{2}$, ${\mathrm{NO}}_{X}$ and other pollutants from the ports of China’s FTZs have decreased year by year. The port environment has gradually improved, and the concept of green development has been thoroughly implemented. However, there are some heterogeneous characteristics in pollutant emissions and their changing trends, and the pollutant emission of coastal ports and eastern ports account for the vast majority. In the downward trend, the reduction of ${\mathrm{NO}}_{X}$ emission was generally higher than that of ${\mathrm{SO}}_{2}\;$emission.

Secondly, both the green TFP and traditional TFP of FTZ ports showed an upward trend, with an average annual growth rate of 4.43% and 1.25%, respectively. It can be seen that the introduction of environmental factors has a significant impact on the estimation of TFP in FTZ ports, which means the non-introduction of environmental factors will lead to the overall underestimation of TFP. The main source of traditional TFP growth is technical efficiency, whereas for green TFP it is technical progress. There is still a large space to further improve the green TFP of FTZ ports through the improvement of technical efficiency.

Thirdly, the growth of green TFP in FTZ ports has great port heterogeneity. Nanjing port has the highest growth rate of green TFP, with an average annual growth rate of 21.95%. Ningbo Port ranked 14th, with an average annual growth rate of 5.46%. At the end of the list, the green TFP of Fuzhou Port showed negative growth. After the introduction of environmental factors, the green TFP of most ports (except for Fuzhou port, Harbin Port and Yangpu Port) increased, compared with the traditional TFP, and the main source of growth changed from technical efficiency without the introduction of environmental factors, to technical progress. The conclusion was the same as the overall time series conclusion.

Fourthly, the growth of ports green TFP in FTZs had great type and regional heterogeneity. Under the classification standard, the annual growth rate of green TFP in inland ports was significantly higher than that in coastal ports. According to the three regional classification standards, the annual growth rate of green TFP from high to low was in the order of: the western region, the eastern region and finally the central region. The central region was more restricted by environmental factors. After the introduction of environmental factors, the TFP growth rate of the central region was lower than that of the eastern and western regions.

Fifthly, from the evolution trend of the internal differences of green TFP between the two types of ports, the differences of green TFP between inland ports and coastal ports are shrinking, and there may be σ convergence. The difference between the two has also narrowed. From the evolution trend of the internal differences of green TFP in the three regional ports, only the differences of green TFP in the central and eastern ports have narrowed, which may exist due to σ convergence. The differences among the three regions are also narrowing.

Based on the above research conclusions, the main enlightenment of this paper is as follows.

Firstly, under the background of economic globalization and regional economic integration, as one of the cores of FTZs, ports in FTZs should pay more attention to the positive impact of TFP on the green and sustainable development of FTZs, and incorporate environmental factors into the evaluation and management system of port TFP. They should also promote the transformation of port growth mode from factor driven to green TFP driven, and further promote the green development of ports in FTZs.

Secondly, it is necessary to maintain the contribution level of technological progress of the TFP of the ports in FTZs, as well as continuously improve the independent innovation ability and technical level of the ports, strictly control pollution emissions, build ecological green ports and strive to improve their core competitiveness. At the same time, the green TFP growth can be promoted through the improvement of technical efficiency, reasonable planning of the port layout, and clear port positioning according to their actual situation, in addition to a focus on the construction of professional and large-scale wharfs, and avoiding the repeated construction and redundant investment of ports. At the same time, we should make full use of the policy advantages of FTA to increase port throughput, improving the carrying capacity of wharfs, and further tapping the potential of existing resources and technologies.

Thirdly, although the regional differences of green TFP growth in ports are shrinking, the government should still formulate corresponding regional difference policies according to the factor endowments of various regions, establish a flexible environmental response system to improve the environmental adaptability of the port industry, and guide ports to make full use of and allocate resources effectively. By promoting the research, development and introduction of environmental protection and clean technologies, strengthening regional green technology exchange and cooperation, we can further narrow the regional differences of port’s green TFP growth, and ultimately promote the overall improvement of port’s green TFP in China’s FTZs.

This study is helpful to scientifically understand the growth trend and heterogeneity of the green TFP of ports in China’s FTZs under environmental constraints, and is of great significance in promoting the green development of ports in China’s FTZs. However, this work is not over yet. In the future, we can continue to study it from at least the following three aspects: first, this study can be further connected with the theme of sustainable development. We can perform research on the sustainable development of FTZ ports from the perspective of green TFP, meaning that green TFP will be regarded as one of the important factors affecting the sustainable development of ports. By constructing a model to quantitatively evaluate the impact of green TFP on port sustainable development under environmental constraints, we can then put forward the port emission reduction strategy from the perspective of sustainable development. Second, we can perform research on the influencing factors of green TFP of FTZ ports. As an important factor affecting the sustainable development of ports, green TFP is an endogenous variable, determined by a series of economic activities. As a continuation of this study, we can further analyze which factors have an important impact on port green TFP. For example, the city’s green infrastructure and comprehensive development level are closely related to the city’s TFP and sustainable development. The port is located in a city, so we can focus on identifying the causal impact of the city’s green infrastructure and comprehensive development level on the port’s green TFP. Third, we can scientifically evaluate the green TFP effect of China’s FTZ strategy. Considering that the ports studied in this paper belong to the ports in China’s FTZs, there may be differences in policy impact between them and non-FTZ ports. Therefore, we can take the establishment of China’s FTZs as a quasi-natural experiment, and scientifically evaluate the real effect of the implementation of China’s FTZ strategy on the port pollutant emission and green TFP of FTZs, by using time-varying difference in difference (DID) and other methods.

## Figures and Tables

**Table 1:** Port input-output literature review.

Port’s Own Input-Output Perspective		
Author	Input indicator	Output indicator
Du et al. (2021) [65]	Length of wharf, number of berths for production, amount of loading and unloading equipment	Cargo throughput, container throughput
Zheng and Yang (2021) [66]	Length of wharf, number of berths for production	Cargo throughput, container throughput
Zhu et al. (2021) [67]	Length of wharf, number of berths for production	Cargo throughput, container throughput
Lu and Li (2018) [68]	Length of wharf, number of berths for production	Port throughput
Port Enterprise Operation Perspective		
Author	Input indicator	Output indicator
Kuang (2007) [62]	Main business cost, number of employees, fixed assets	Net profit, main business revenue, earnings per share
Wang and Wu (2016) [63]	Number of employees	Net profit, main business revenue, earnings per share
Feng et al. (2017) [64]	Main business cost, fixed assets	Net profit, main business revenue
Han et al. (2021) [69]	Number of employees, number of bridge cranes	Net profit, main business revenue
Undesirable Output Perspective		
Author	Input indicator	Output indicator
Liu et al. (2021) [56]	Number of berths for production, length of wharf, number of employees, net assets	Port throughput, net profit, SO_2_, NO_x_emissions
Liu and Wang (2018) [70]	Length of berths for production, energy consumption (standard coal), production berth above 10,000 tons	Container throughput, CO_2_ emissions
Luo et al. (2014) [54]	Berth length, port area, shore jib crane, pusher crane	Container throughput, CO_2_ emissions
Sun et al. (2017) [71]	Number of employees, fixed asset, operating costs	Cargo throughput, net profit, NO_X_ emissions
Wang and Liang (2018) [72]	Number of berths for production, number of employees, oil consumption, electric consumption	Cargo throughput, CO_2_ emissions

**Table 2:** Descriptive statistical results of input-output variables.

Variable Type	Variable Name	Unit	Obs.	Mean	Median	S.D.	Min	Max
“Good output”	Cargo Throughput Container throughput	10,000 Tons 10,000 TEUs	196 196	21,793 675	14,236.5 136.54	19,223.41 1441.51	348 0	70,542 16,602
“Bad output”	SO_2_ NO_x_	Ton Ton	196 196	2097 3281	187.64 1555.95	3499.4 4780.1	1.98 19.68	17,028 24,075
Ports investment	Number of berths for production	Unit	196	225	151	231.39	22	1402
	Length of Wharf	Meter	196	25,585	22,674	18,892	2084	76,469

**Table 3:** Port TFP index and its decomposition term (2011–2017).

Year	No Environmental Factors Were Introduced			Environmental Factors Were Introduced		
	TEC	TPC	TFPC	TEC	TPC	TFPC
2012	1.0560	0.8232	0.8693	0.9385	0.9271	0.8701
2013	1.0570	1.0161	1.0740	1.0064	1.0965	1.1035
2014	1.0414	0.9765	1.0170	1.1400	0.9005	1.0265
2015	1.1115	0.9140	1.0159	0.9952	1.0254	1.0205
2016	1.0200	0.9856	1.0054	0.9392	1.1678	1.0968
2017	1.0733	1.0350	1.1109	1.0280	1.1439	1.1758
Mean value	1.0595	0.9556	1.0125	1.0057	1.0384	1.0443

**Table 4:** TFP index of each port and its decomposition term (2011–2017).

Port	Type	Region	No Environmental Factors Were Introduced				Environmental Factors Were Introduced			
			TEC	TPC	TFPC	TFP Ranking	TEC	TPC	TFPC	TFP Ranking
Changsha	inland	central	1.1822	0.9621	1.1374	2	1.1098	1.0336	1.1471	4
Dalian	coastal	eastern	1.0602	0.9615	1.0194	13	0.9255	1.1061	1.0237	18
Fuzhou	coastal	eastern	0.8200	0.9616	0.7885	28	0.7870	0.9999	0.7869	28
Guangzhou	coastal	eastern	1.0887	0.9607	1.0459	8	1.0754	1.0333	1.1112	9
Harbin	inland	central	1.0011	0.9615	0.9625	23	0.9317	1.0297	0.9593	25
Haikou	coastal	eastern	0.9682	0.9620	0.9313	27	1.0000	1.0000	1.0000	20
Hangzhou	inland	eastern	1.2062	0.9615	1.1597	1	1.1301	1.0325	1.1668	2
Hefei	inland	central	0.9880	0.9624	0.9508	24	0.9429	1.0332	0.9742	24
Lianyungang	coastal	eastern	1.0275	0.9645	0.9910	19	0.9496	1.0567	1.0034	19
Luzhou	inland	western	1.1632	0.9619	1.1188	3	1.0043	1.1199	1.1248	6
Nanjing	inland	eastern	1.1301	0.9620	1.0872	5	1.1790	1.0343	1.2195	1
Nanning	inland	central	1.0429	0.9615	1.0027	18	1.0299	1.0228	1.0533	15
Ningbo	coastal	eastern	1.0782	0.9437	1.0175	14	1.0555	0.9991	1.0546	14
Qinzhou	coastal	central	0.9802	0.9626	0.9435	26	0.8180	1.1619	0.9504	26
Qingdao	coastal	eastern	1.0209	0.9661	0.9863	20	1.0000	1.0000	1.0000	21
Shanghai	coastal	eastern	1.1261	0.9150	1.0304	11	1.1188	1.0014	1.1204	7
Shenzhen	coastal	eastern	1.1795	0.8584	1.0125	16	1.0000	1.0712	1.0712	11
Suzhou	inland	central	1.0674	0.9623	1.0271	12	1.0000	1.0323	1.0323	17
Tangshan	coastal	eastern	1.0000	0.9802	0.9802	21	1.0000	1.0000	1.0000	22
Tianjin	coastal	eastern	1.0463	0.9684	1.0132	15	1.0315	1.0044	1.0361	16
Wuhu	inland	central	1.0908	0.9625	1.0499	7	1.0142	1.0415	1.0564	13
Wuhan	inland	central	1.0967	0.9620	1.0551	6	1.0413	1.0359	1.0787	10
Xiamen	coastal	eastern	1.0833	0.9298	1.0073	17	1.0158	1.1244	1.1422	5
Yantai	coastal	eastern	1.0113	0.9623	0.9731	22	0.9726	1.0066	0.9791	23
Yangpu	coastal	eastern	0.9825	0.9622	0.9454	25	0.9110	1.0089	0.9191	27
Yueyang	inland	central	1.0743	0.9623	1.0338	10	1.0000	1.0663	1.0663	12
Chongqing	inland	western	1.1545	0.9625	1.1112	4	1.1191	1.0342	1.1574	3
Zhuhai	coastal	eastern	1.0839	0.9618	1.0425	9	1.1066	1.0084	1.1159	8
Mean value			1.0595	0.9556	1.0125		1.0057	1.0384	1.0443	

**Table 5:** TFP index of port type and region and its decomposition mean (2011–2017).

Port	No Environmental Factors Were Introduced			Environmental Factors Were Introduced		
	TEC	TPC	TFPC	TEC	TPC	TFPC
Overall average	1.0595	0.9556	1.0125	1.0057	1.0384	1.0443
Inland	1.0977	0.9620	1.0561	1.0393	1.0427	1.0837
Coastal	1.0317	0.9508	0.9810	0.9812	1.0352	1.0157
Eastern	1.0509	0.9520	1.0005	1.0103	1.0282	1.0388
Central	1.0551	0.9621	1.0151	0.9824	1.0523	1.0337
Western	1.1588	0.9622	1.1150	1.0601	1.0762	1.1410

## Data Availability

The data presented in this study are available on request from the corresponding author (coldxu520@163.com).
